# Genome sequence data announcement of *Bifidobacterium bifidum* strain ICIS-202 isolated from a healthy human intestine stimulating active nitrogen oxide production in macrophages

**DOI:** 10.1016/j.dib.2019.104761

**Published:** 2019-11-06

**Authors:** Oleg V. Bukharin, Sergey V. Andryuschenko, Natalia B. Perunova, Elena V. Ivanova, Irina N. Chainikova, Irina A. Zdvizhkova

**Affiliations:** Orenburg Federal Research Center of the Ural Branch of the Russian Academy of Sciences, Institute for Cellular and Intracellular Symbiosis, Orenburg, Russia

**Keywords:** WGS, Bifidobacterium, Nitrogen oxide, Cytokines, Human gut, Microbiocoenosis

## Abstract

This report presents the data on the draft genome sequence of *Bifidobacterium bifidum* strain ICIS-202. The strain, isolated from the intestine of a young healthy woman, was deposited in the State Collection of Microorganisms of Normal Microbiota in Gabrichevsky Institute of Epidemiology and Microbiology, Moscow, Russian Federation as a prospective candidate for probiotic development. The size of the genome was 2,265,060 bp (62,4% G + C content). The annotation revealed 1771 coding sequences, including 1771 proteins, 5 rRNA, 52 tRNA, and 3 ncRNA genes. The draft genome sequence data of *B. bifidum* strain ICIS-202 is available in DBJ/EMBL/GenBank under the accession nos. SSMS00000000.1, PRJNA412271 and SAMN07709009 for Genome, Bioproject and Biosample databases, respectively.

**Table:** Specifications Table

Subject area	Biology
More specific subject area	Microbial genomics
Type of data	Genomic sequence, predicted genes and annotation
How data was acquired	Whole genome sequencing of fragment libraries with Illumina MiSeq platform, following *de novo* genomic assembly
Data format	Annotated draft genome assembly
Experimental factors	The genomic DNA extraction, fragment library preparation, Illumina sequencing, *de novo* assembly and annotation procedures
Experimental features	The genomic DNA extraction was performed using the standard phenol-chloroform method; fragment library was prepared by using Illumina, San Diego, CA Kit. The library was sequenced in a 2 × 300-nucleotide run using the MiSeq reagent kit version 3 and MiSeq desktop sequencer (Illumina). The reads were quality trimmed using the sliding window mode of the Trimmomatic program. De novo genome assembly performed using the SPAdes genome assembler (St. Petersburg genome assembler, version 3.10.1)
Data source location	The culture of strain *Bifidobacterium bifidum* strain ICIS-202 is deposited in State Collection of Microorganisms of Normal Microbiota in Gabrichevsky Institute of Epidemiology and Microbiology, Moscow, Russian Federation.
Data accessibility	The draft genome sequence data of strain *Bifidobacterium bifidum* strain ICIS-202 is available in DBJ/EMBL/GenBank under the accession nos. SSMS00000000.1 (https://www.ncbi.nlm.nih.gov/nuccore/SSMS00000000), PRJNA412271(https://www.ncbi.nlm.nih.gov/bioproject/PRJNA412271) and SAMN07709009(https://www.ncbi.nlm.nih.gov/biosample/SAMN07709009) for Genome, Bioproject and Biosample, respectively.
Related research article	Andryuschenko SV, Ivanova EV, Perunova NB, Zdvizhkova IA, Bekpergenova AV, Bukharin OV. Draft Genome Sequence of *Bifidobacterium bifidum* Strain ICIS-310, Isolated from the Feces of a Healthy 5-Year-Old Child from Orenburg, Russia. Microbiol Resour Announc. 2018 Nov 8; 7 (18). pii: e01271-18. https://doi.org/10.1128/MRA.01271-18.

**Table:** 

**Value of the Data** • Initial microbiological tests showed the ability of this strain to stimulate active nitrogen oxide compound production in macrophages. The genome's draft assembly makes it possible to search for new proteins and ways for the biosynthesis of secondary metabolites. • Draft genome data may be used by scientific community, which carry out research in the field of discovering molecular mechanisms in probiotic production and activity. • Draft genome data may be used to broaden the knowledge on phylogenetic diversity of Bifidobacteria and, specifically, Bifidobacterium bifidum species.

Bifidobacteria are predominant in the fecal microbiota of infants, and they are considered to be the most important physically and mentally beneficial bacteria for infants [1]. Infants with bifidobacteria-dominated gastrointestinal tracts are more resistant to colonization by pathogens, respond better to some vaccines, and possess better-functioning gut barriers [[2], [3], [4], [5]]. Bifidobacteria also appears to enhance immune surveillance and reduce inflammation at the same time [[5], [6], [7], [8]]. The presence of the infant-type bifidobacteria when the weaning takes place may help in guiding the immune system towards the tolerance during the introduction of new foods and their associated antigens, potentially influencing the development of allergic diseases [[9], [10], [11], [12]]. Among other motivations, bifidobacterial levels have been studied in infants across the globe for these public health reasons.

The bacterial supernatant is a dynamic entity, which depends on many components and plays an essential role in immune response modulation. The aim was to investigate the bifidobacteria strain's effect on phagocytic system cells (macrophages).

## 1 Data

The strain ICIS-202 was initially identified as B. bifidum with the biochemical species identification kit ANAEROtest 23 (Lachema, Czech Republic). It revealed, that the ICIS-202 strain ferments glucose, fructose, galactose, lactose, sucrose, raffinose, arabinose, urease and xylose. The disc diffusion test revealed antibiotic resistance of the B. bifidum strain ICIS-202 to kanamycin, cefloxacin, ciprofloxacin, lomefloxacin.

We conducted studies on (CBAxC57B16)F1/c line mice 18–20 g in weight using the bacterial supernatant of B. bifidum strain ICIS-202. The macrophages, obtained from the mice's peritoneal cavity, were individually cultivated with the *B. bifidum* strain ICIS-202's culture medium supernatant for evaluation of its effect on nitric oxide production in the macrophages. The bifidobacteria's supernatant displayed a stimulating effect on ability to produce NO/NO_2_ of macrophages in vitro. Incubation of peritoneal macrophages with the bifidobacteria's supernatant was accompanied by a moderate increase in the basic production of NO/NO_2_ compared with the control. The effect of *B. bifidum* strain ICIS-202's culture medium on nitrogen oxides and cytokine production by mouse macrophages is presented in Table 1.

**Table 1:** Secretion of nitrogen oxide and cytokines by mouse peritoneal macrophages after incubation with B. bifidum strain ICIS-202 culture medium.

	NO/NO_2_, μmol/l	IFN-γ, pg/ml	TNF-α, pg/ml	IL-8, pg/ml	IL-1β, pg/ml	IL-10, pg/ml	IL-4, pg/ml
Peritoneal macrophages (control)	55,8 (54,2–56,7)	15,3 (14,9–15,6)	13,8 (12,9–14,8)	23,0 (21,2–25,1)	88,8 (85,6–96,7)	14,7 (12,2–16,7)	0,7 (0,64–0,75)
Peritoneal macrophages + B. bifidum ICIS-202 culture medium (experimental)	164,8 (160,2–177,4)	30,8 (54.2–56.7)	15,0 (14,2–15,6)	22,7 (21,1–23,4)	10,6 (8,5–13,2)	45,6 (44,1–46,8)	0,8 (0,72–0,86)

## 2 Experimental design, materials and methods

Preparation of DNA libraries and sequencing were performed in the Center of Shared Equipment “Persistence of microorganisms” of the Institute for Cellular and Intracellular Symbiosis of the Ural Branch of the Russian Academy of Sciences (RAS; Orenburg, Russia).

The B. bifidum strain ICIS-202 was cultivated in 4 ml of Schaedler medium (HiMedia Laboratories Pvt. Limited) during 48 hours at 0.6% oxygen and 5% carbon dioxide atmosphere with temperature of 37 C in CO_2_-incubator (BINDER, Tuttlingen, Germany). After incubation the culture was centrifuged at 4000 g for 6 min. The sediment was suspended again in 50 μl of tris-buffered saline with 2 μg of hen egg white lysozyme (HEWL), and then incubated at 37 C during 1 hour. After that, the suspension was mechanically homogenized by 1.4 mm silica beads at 6.5 m/s speed for 1 min. The DNAses were inactivated by heating the suspension up to 95 C for 10 minutes; then 50 μl of 10% SDS solution and 2 μl of 100 mg/ml proteinase K solution were added to the suspension with subsequent incubation at 60 C for 60 min. The extracted DNA solution was purified using the standard phenol-chloroform extraction method (6), and precipitated with ethanol (7). The DNA sediment was dissolved in 30 μl of «Milli-Q» deionized water.

Extracted DNA of the B. bifidum strain ICIS-202 was used to prepare a DNA library by using the Nextera XT DNA sample preparation kit (Illumina, San Diego, CA). The library was sequenced in a 2 × 300-nucleotide run using the MiSeq reagent kit version 3 and MiSeq desktop sequencer (Illumina). The reads were quality trimmed using the Trimmomatic program's sliding window mode (15). De novo genome assembly performed using the SPAdes genome assembler (St. Petersburg genome assembler, version 3.10.1) (8). The total of 2,265,060 bp with an N50 of 357,567, a G + C content of 62,4%, with an average coverage of 42,1. It was removed from the analysis. The genome sequence was annotated using the National Center for Biotechnology Information (NCBI) Prokaryotic Genome Annotation Pipeline (PGAP) (http://www.ncbi.nlm.nih.gov/genome/annotation_prok), which revealed 1771 protein-coding gene sequences, 110 pseudogenes, 5 rRNA genes (5S, 16S, and 23S), 52 tRNA genes, and 3 noncoding RNA (ncRNAs) genes.

The culture medium's supernatant of studying strain ICIS-202 was collected using high speed centrifugation (at 4000 *g* for 15 min) after incubation under the aforementioned conditions, and dialyzed on a 10 kDa membrane.

Nitrogen oxide production studies were performed on macrophages from mice strain (CBAxC57Bl6)F1/c, obtained from the animal nursery of the Branch “Stolbovaya” of the Scientific Center for Biomedical Technologies of the Federal Medical and Biological Agency of Russia. All studies were performed in complying with the European Convention's rules for the vertebrate animals protection used for research and other scientific purposes (March 18, 1986) and the “Rules of laboratory practice in the Russian Federation” Order of the Ministry of Health of the Russian Federation N.267 dated June 19, 2003 [13]. The mice were injected intraperitoneally with sterile Brewer-thioglycollate medium (2 mL, 4% w/v) to recruit macrophages to peritoneal cavity. Four days after injection, the mice were sacrificed for macrophage collection from mouse peritoneal cavity using cold PBS. The cell pellets was obtained after centrifugation at 2000 rpm for 5 min. The cells will be incubated in DMEM supplemented with 10% FBS and 1% gentamicin (LLC RLS-Patent, Russia) for 2 h in CO_2_-incubator (BINDER, Tuttlingen, Germany) and washed three times to remove non-adherent cells [14]. Isolated macrophages secreted high levels of IFN-γ, IL-8, TNF-α, IL-1β and low levels of IL-10, IL-4 which allowed them to be classified as pro-inflammatory macrophages (M1-like). Cytokine level in the cultural medium of the macrophages was determined by enzyme immunoassay by using the appropriate test systems (Bender MedSystems, Austria).

Macrophages were stimulated by culture medium's supernatant *B. bifidum* strain ICIS-202 in the 1:100 cell ratio in 96-well plates for 24 hours at 37 C in an atmosphere of CO_2_ (5%). Upon finishing, supernatants were harvested and stored at −20 C.

The nitric oxide's concentration level in the cultural medium of the macrophages was determined by test systems Total NO/Nitrite/Nitrate Assay (R&D Systems, Inc., USA). The differences between the control and test were considered statistically meaningful at p < 0.05.

## Funding statement

This work was carried out in the framework of fundamental research at the Ural Branch of the Russian Academy of Sciences (project 18-7-8-34). The reported study was funded by RFBR according to the research projects 19-415-560002 and 18-34-00853.
